# Mechanisms of glycine formation in cold interstellar media: a theoretical study

**DOI:** 10.1098/rsos.231957

**Published:** 2024-05-22

**Authors:** Pannipa Panajapo, Parichart Suwannakham, Phorntep Promma, Kritsana Sagarik

**Affiliations:** ^1^ School of Chemistry, Institute of Science, Suranaree University of Technology, Nakhon Ratchasima 30000, Thailand

**Keywords:** glycine, formation, mechanisms, CASPT2, TD-DFT lowest singlet-excited state, transition state theory

## Abstract

The possibility of the formation of glycine (Gly) from fundamental gas molecules in cold interstellar media was studied using quantum chemical methods, transition state theory and microcanonical molecular dynamics simulations with surface hopping dynamics (NVE-MDSH). This theoretical study emphasized five photochemical pathways in the lowest singlet-excited (*S*_1_) state, thermochemical processes after non-radiative *S*_1_→*S*_0_ relaxations, and photo-to-thermal energy conversion in the NVE ensemble. The optimized reaction pathways suggested that to generate a reactive singlet dihydroxy carbene (HOCOH) intermediate, photochemical pathways involving the H_2_O…CO van der Waals and H_2_O−OC hydrogen bond precursors (Ch (1)_Step (1)) possess considerably lower energy barriers than the *S*_0_ state pathways. The Gibbs free energy barriers (∆*G* **^ǂ^**) calculated after the non-radiative *S*_1_ *→S*_0_ relaxations indicated higher spontaneous temperatures (*T*_s_) for the formation of the HOCOH intermediate (Ch (1)_Step (1)) than for Gly formation (Ch (1)_Step (2) and Ch (4)). Although the termolecular reaction in Ch (4) possesses a low energy barrier, and is thermodynamically favourable, the high exothermic *S*_1_ *→S*_0_ relaxation energy leads to the separation of the weakly associated H_2_O…CH_2_NH…CO complex into single molecules. The NVE-MDSH results also confirmed that the molecular processes after the *S*_1_ *→S*_0_ relaxations are thermally selective, and because the non-radiative *S*_1_ *→S*_0_ relaxation temperatures are exceedingly higher than *T*_s_, the formation of Gly on consecutive reaction pathways is non-synergistic with low yields and several side products. Based on the theoretical results, photo-to-thermal control strategies to promote desirable photochemical products are proposed. They could be used as guidelines for future theoretical and experimental research on photochemical reactions.

## 1. Introduction

The photochemistry of small molecules is fundamental to understanding photochemical reactions in the Earth’s atmosphere and interstellar media [1]. The mechanisms for the formation of amino acids through photochemical processes have received special attention in recent decades because experiments have shown that they can be generated in cold interstellar media through UV irradiation [2,3]. The reactions generally involve reactive intermediates, which cannot be detected easily in experiments [4–6]. As a small proteinogenic molecule, glycine (NH_2_CH_2_COOH, abbreviated Gly) has been frequently chosen as a model molecule for studying the photochemistry of polypeptides [7]. As the smallest amino acid found in meteorites [8], attempts have been made to synthesize Gly under extraterrestrial [2] and primitive Earth conditions from fundamental gases such as H_2_, CO, H_2_O, NH_3_ and CH_4_. The Strecker synthesis [9] and Miller–Urey experiments [10] are well-known classical examples used to study prebiotic chemistry and the origin of life. In the Miller–Urey prebiotic experiment, more than 20 different amino acids were produced in continuous electrical sparks caused by a pair of electrodes.

**Figure 1.**
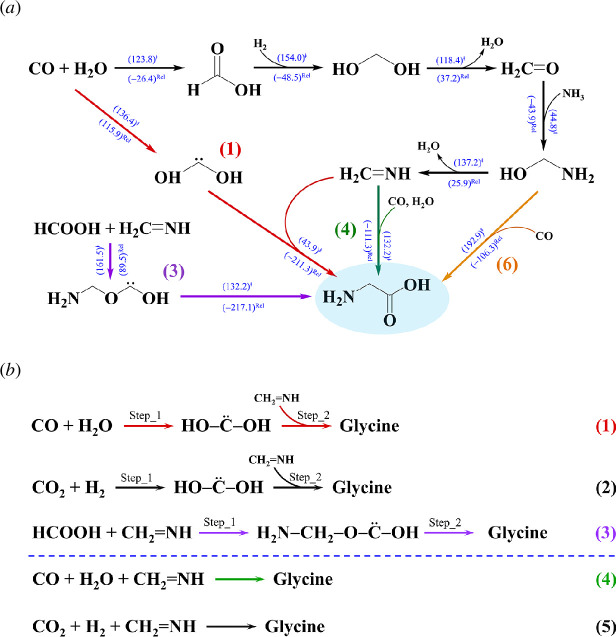
(*a*) Elementary reactions for Gly formation from gas molecules in the electronic ground (*S*_0_) state suggested from *ab initio* nanoreactor simulations [11]. (*b*) Bimolecular (Ch (1)−(3)) and termolecular reaction mechanisms (Ch (4)−(5)) for Gly formation from gas molecules. (…)^ǂ^ = energy barrier in kJ mol^−1^; (…)^Rel^ = relative energy with respect to the transition structure in kJ mol^−1^.

To study the multiple Gly synthesis pathways in the Miller–Urey experiment, the so-called ‘*ab initio* nanoreactor’ was used [11]. The theoretical method is based on *ab initio* molecular dynamics (AIMD) simulations on reacting molecules and analysis and refinements to suggest an accurate reaction network. The *ab initio* method was conducted in the electronic ground (*S*_0_) state using the Hartree–Fock (HF) method with the 3-21G basis set (abbreviated HF/3-21G). The minimum energy pathways obtained based on AIMD simulations indicated that in the *S*_0_ state, a significant fraction of the elementary reactions takes place with energy barriers (Δ*E* **^ǂ^**) less than 209 kJ mol^−1^, and Gly could be formed via four different pathways, in which singlet dihydroxy carbene (HOCOH), methanimine (CH_2_NH) and methanolamine (HOCH_2_NH_2_) play an important role, e.g. in channels (1), (3) and (6), in figure 1*a*, hereafter abbreviated Ch (1), Ch (3) and Ch (6), respectively.

The energy refinements using the density functional theory (DFT) method with the 6-31+G(d,p) basis set (DFT/6-31+G(d,p)) and intrinsic reaction coordinate (IRC) method [11] revealed that in the *S*_0_ state, the consecutive reactions involving the formation of methanolamine/aminomethanol (HOCH_2_NH_2_) in Ch (6) possess the highest energy barrier, Δ*E* **^ǂ^** = 193 kJ mol^−1^, whereas the consecutive reactions in Ch (1), which involve the formation of HOCOH directly from CO and H_2_O (abbreviated Ch (1)_Step (1) and Ch (1)_Step (2) in figure 1*b*), possess considerably lower energy barriers, Δ*E* **^ǂ^** = 136 and 44 kJ mol^−1^ [11], and the termolecular reaction in Ch (4) possesses Δ*E* **^ǂ^** = 132 kJ mol^−1^.

Alternative bimolecular and termolecular reactions to generate Gly in the *S*_0_ state were proposed in [12], in which the precursors were suggested to be CO_2_ and H_2_, and CO_2_, H_2_ and CH_2_NH in Ch (2) and (5) in figure 1*b*, respectively. Based on the *S*_0_ potential energy curves computed using the DFT/6-31++G(d,p) method with zero-point vibrational energy corrections (ZPC), formation of the HOCOH intermediate (Ch (2)_Step (1)) possesses a high energy barrier, whereas the reaction between HOCOH and CH_2_NH to generate Gly (Ch (2)_Step (2)) is almost barrierless. In contrast, while the concerted termolecular reaction (CO, H_2_O and CH_2_NH) in Ch (4) proceeds on a low energy barrier path [11], the termolecular reaction (CO_2_, H_2_ and CH_2_NH) in Ch (5) possesses a considerably higher energy barrier with a possibility for quantum mechanical tunnelling of the hydrogen atom.

In our previous study [13], photodissociations of Gly in the lowest singlet-excited (*S*_1_) state were theoretically studied using the DFT, time-dependent DFT (TD-DFT) and complete active space second-order perturbation theory (CASPT2) methods with the aug-cc-pVDZ basis set and microcanonical molecular dynamics simulations with surface hopping dynamics (NVE-MDSH). This theoretical study emphasized unimolecular dissociation and isomerization and non-radiative *S*_1_→*S*_0_ relaxations that generate molecular products in cold interstellar media. The results showed that the high exothermic *S*_1_→*S*_0_ relaxation temperature could lead to at least eight molecular product channels, in which the vertically excited Gly was primarily dissociated and isomerized, leading to reactive and stable molecules in different temperature ranges. The thermally selective reactions favoured the lowest energy conformer structure Ip as the precursor. These theoretical results showed in detail for the first time how non-radiative *S*_1_→*S*_0_ relaxations generate molecules in cold interstellar media without heat transfer from the surroundings.

Because the reported energy barriers for the formation of Gly in the *S*_0_ state are in our opinion too high to occur in cold interstellar media [11], and our previous studies showed that reactive intermediates can be effectively generated through photochemical processes in the *S*_1_ state, from which molecular products can be subsequently formed in the *S*_0_ state, an attempt was made in this work to search for low energy barrier photochemical pathways for Gly formation. The theoretical study began with the *S*_0_→*S*_1_ vertical excitation of fundamental gas precursors in the five reaction channels (Ch (1)−(5) in figure 1*b*), for which non-radiative photochemical pathways to generate intermediates in the *S*_1_ state were primarily studied using the DFT/B3LYP/aug-cc-pVDZ, TD-DFT/B3LYP/aug-cc-pVDZ and nudged elastic band (NEB) methods. Because the DFT and TD-DFT methods are based on a single-reference wavefunction approximation and multiconfigurational characteristics of covalent bond dissociation and formation could be important, the structures at the *S*_0_/*S*_1_ intersection were refined using the state average-coupled perturbed multiconfigurational self-consistent field (SA-CPMCSCF/6-31G(d)) method, from which the non-radiative *S*_1_→*S*_0_ relaxations of the intermediates and formations of Gly and side products were studied in the *S*_0_ state using the DFT/B3LYP/aug-cc-pVDZ method. The performance of the DFT and TD-DFT methods was also assessed using the CASPT2 method.

Because formations of reactive intermediates in the *S*_1_ state are usually ultrafast [4–6,13], the kinetic and thermodynamic properties were studied only after the non-radiative *S*_1_→*S*_0_ relaxations using the transition state theory (TST) method. The dynamics and mechanisms for the formation of Gly and side products were further studied using NVE-MDSH simulations, from which the role played by the exothermic *S*_1_→*S*_0_ relaxation energy and the interplay between the photo and thermal energies were analysed and discussed in detail.

## 2. Computational methods

### 2.1. Quantum chemical calculations

To study the structures and energetics of the precursors in the *S*_0_ and *S*_1_ states, DFT and TD-DFT methods with the Becke, 3-parameter, Lee–Yang–Parr (B3LYP) hybrid functional and aug-cc-pVDZ basis set were used. For the TD-DFT method, the Tamm–Dancoff approximation was applied to avoid singlet instabilities in the *S*_1_ state calculations [14]. Although the DFT/B3LYP/aug-cc-pVDZ and TD-DFT/B3LYP/aug-cc-pVDZ methods had been systematically tested and were applied successfully in our previous work on Gly [13], they were assessed again in this study using the CASPT2/aug-cc-pVDZ method. The active spaces used in the CASPT2 calculations are summarized in electronic supplementary material, table S1. Both DFT and TD-DFT calculations were made using the TURBOMOLE 7.50 software package [15]. The state-averaged CASPT2 calculations were performed in the *S*_0_ and *S*_1_ states with equal weights using the MOLPRO software package [16,17], for which the Werner–Mayer–Knowles nonlinear method was used in orbital/state optimizations [18–20].

#### 2.1.1. Geometry optimizations

Because our previous study [13] showed that photochemical products depend strongly on the structures of the vertically excited precursors in the *S*_0_ state and the structures at the *S*_0_/*S*_1_ intersection and because fundamental gases such as H_2_, CO, CO_2_ and H_2_O were suggested to be involved in Gly formation (Ch (1)−(5)) [11], their equilibrium structures in the *S*_0_ state and *S*_0_→*S*_1_ vertical excitation energies (Δ*E* ^Ex^) were calculated using the DFT/B3LYP/aug-cc-pVDZ and TD-DFT/B3LYP/aug-cc-pVDZ methods, respectively. Based on the proposed mechanisms in [11,12] (figure 1*b*), the equilibrium structures of the van der Waals (VdW) and hydrogen bond (H-bond) precursors and intermediates in Ch (1)−(5) were also optimized in the *S*_0_ state. To assess the DFT method, the optimized structures were reoptimized using the CASPT2 method.

#### 2.1.2. Reaction pathway optimizations

Because the photochemical pathways that generate Gly in cold interstellar media were hypothesized in this work to involve the *S*_0_→*S*_1_ vertical excitation of the precursors (binary or ternary complexes formed from fundamental gases), non-radiative *S*_1_→*S*_0_ relaxations of the excited precursors, and generation of the intermediates/products in the *S*_0_ state, reaction pathway optimizations were performed for Ch (1)−(5) in both *S*_1_ and *S*_0_ states using the NEB method [21]. The NEB potential energy curves were computed using limited-memory Broyden–Fletcher–Goldfarb–Shanno (LBFGS) optimizers included in the ChemShell software package [22].

In this work, to verify the results obtained from the TD-DFT and DFT methods, the *S*_1_ and *S*_0_ potential energy curves were compared with CASPT2 calculations with the geometries obtained from the TD-DFT and NEB methods. The structures at the *S*_0_/*S*_1_ intersection were confirmed using the SA-CPMCSCF/6-31G(d) conical intersection optimization method (electronic supplementary material, table S2). A schematic diagram showing step-by-step non-radiative photochemical pathway optimizations and steps to optimize the reaction pathways for the formation of the HOCOH intermediate from the H_2_O…CO VdW precursor in Ch (1)_Step (1) are explained in detail in the electronic supplementary material. In this work, to discuss the reaction pathways, […]^eq^ and […] denote the equilibrium structures and structures on the *S*_0_ potential energy curves, respectively, whereas […]^*^ are the structures on the *S*_1_ potential energy curves. The transition structures and structures at the *S*_0_/*S*_1_ intersection are […]^ǂ^ and […]^§^, respectively.

### 2.2. Transition state theory calculations

Because kinetic and thermodynamic properties were not reported in all previous quantum chemical studies, the rate constants, enthalpies and Gibbs free energies for the formation of Gly and side products were calculated in this work using the TST method [21,23,24]. The calculations of the kinetic and thermodynamic properties using the TST method are discussed in [25] and in the electronic supplementary material (tables S3−S5).

#### 2.2.1. Kinetics of reaction pathways

The classical (*k* ^Class^) and quantized-vibrational (*k* ^Q-vib^) rate constants [26] were primarily computed over the temperature range of 100–4500 K. *k* ^Q-vib^ was computed using the ZPC barrier obtained by including the zero-point correction energy (Δ*E* ^ZPE^) to the energy barrier obtained from the NEB method (Δ*E* ^ǂ^). To study the effects of quantum mechanical tunnelling on the reaction rates, the crossover temperatures (*T*_c_) were computed; for the pathways that involve proton/H-atom transfer, quantum mechanical tunnelling could dominate below *T*_c_ [27,28]. In addition, the simple Wigner-corrected rate constants (*k* ^S-Wig^) were calculated, for which *k* ^S-Wig^ = *k* ^Q-vib^ at the classical limit (*ħ* = 0) [29].

#### 2.2.2. Thermodynamics of reaction pathways

The activation free energies (ΔG^ǂ^), enthalpies (Δ*H* **^ǂ^**) and entropies (Δ*S* **^ǂ^**) of the elementary reactions were computed from the rate constants (*k* ^Q-vib^) obtained from the TST method. All the kinetic and thermodynamic calculations were performed using the DL-FIND program [30] included in the ChemShell package [22].

**Figure 2.**
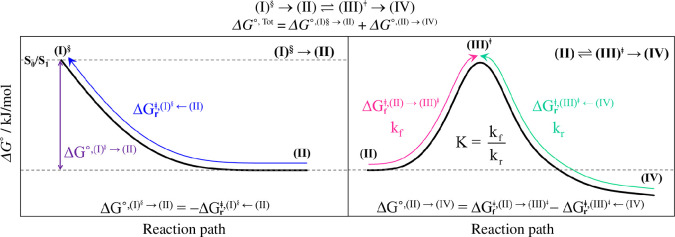
Calculations of Gibbs free energies on the reaction pathways without ((I)^§^→(II)) and with energy barrier ((II)⇌(III)^ǂ^→(IV)). ∆*G*˚^,Tot^ = total Gibbs free energy change of the (I)^§^→(II)⇌(III)^ǂ^→(IV) reaction; ${\Delta G}_{r}^{ǂ,{\left(I\right)}^{§}\leftarrow (II)}$ = Gibbs free energy barrier for (I)^§^←(II); $\Delta G^{\circ ,(I{)}^{\circ }\to (II)}$ = $-\Delta G^{\circ ,(I{)}^{§}\leftarrow (II)}$ = Gibbs free energy change for (I)^§^→(II); ${\Delta G}_{f}^{ǂ,(II)\to {\left(III\right)}^{ǂ}}$ = Gibbs free energy barrier for (II)⇌(III)^ǂ^; ${\Delta G}_{r}^{ǂ,{\left(III\right)}^{ǂ}\leftarrow (IV)}$ = Gibbs free energy barrier for (III)^ǂ^←(IV); *K* = equilibrium constant; f and r = forward and reverse reactions.

The total Gibbs free energy changes after the exothermic *S*_1_→*S*_0_ relaxation (∆*G*˚^,Tot^) were calculated over the temperature range of 100−4500 K, for which two types of reaction pathways were considered, namely the *S*_1_→*S*_0_ relaxation on a barrierless potential ((I)^§^→(II)) and reaction pathway with an energy barrier ((I)^§^→(II)⇌(III)^ǂ^→(IV)) as shown in figure 2, regarded as single-step and consecutive reaction pathways, respectively. ∆*G*˚^,Tot^ for both types of elementary reactions were computed using ∆*G* ^ǂ^ obtained from the TST method. For (I)^§^→(II), $\Delta G^{˚,{\left(I\right)}^{§}\to (II)}$ = $-\Delta G_{r}^{‡,(I{)}^{§}\leftarrow (II)}$ , whereas ∆*G*˚^,(II)→(IV)^ = ${\Delta G}_{f}^{ǂ,(II)\to {\left(III\right)}^{ǂ}}$ − ${\Delta G}_{r}^{ǂ,{\left(III\right)}^{ǂ}\leftarrow (IV)}$ for (II)⇌(III)^ǂ^→(IV) and ∆*G*˚^,Tot^ = $\Delta G^{˚,{\left(I\right)}^{§}\to (II)}$ + ∆*G*˚^,(II)→(IV)^ for (I)^§^→(II)⇌(III)^ǂ^→(IV).

For consecutive reaction pathways, because non-radiative *S*_1_→*S*_0_ relaxations are exothermic (∆*H*˚< 0 with ${\Delta G}_{r}^{ǂ,(I{)}^{§}\leftarrow \left(II\right)}$ > 0), the thermodynamic spontaneity is temperature dependent. For the exothermic *S*_1_→*S*_0_ relaxation followed by the reaction with an energy barrier (I)^§^→(II)⇌(III)^ǂ^→(IV), the spontaneous temperature (*T*_s_), below which the transition structure (III)^ǂ^ is spontaneously formed from (I)^§^, was obtained from the plots of $\Delta G_{r}^{‡,(I{)}^{§}\leftarrow (II)}$ and ${\Delta G}_{f}^{ǂ,(II)\to {\left(III\right)}^{ǂ}}$ versus temperature. For example, in figure 2 , (I)^§^→(II)⇌(III)^ǂ^ is spontaneous when ${\Delta G}_{f}^{ǂ,(II)\to {\left(III\right)}^{ǂ}}-{\Delta G}_{r}^{ǂ,(I{)}^{§}\leftarrow \left(II\right)}$ ≤ 0. In other words, formation of (III)^ǂ^ is spontaneous when *T* ≤ *T*_s_.

### 2.3. Surface hopping molecular dynamics simulations

To study the time evolutions of the photochemical pathways suggested based on the NEB and TST methods, NVE-MDSH simulations were performed using the equilibrium structures of the VdW and H-bond precursors. The initial configurations for the *S*_0_→*S*_1_ vertical excitations were generated using the Wigner distribution (*n* = 307), from which NVE-MDSH simulations were performed in the *S*_1_ and *S*_0_ states using the TD-DFT and DFT methods, respectively.

In this work, the NEWTON-X software package [31,32] interfaced with TURBOMOLE 7.50 was used to study the surface hopping dynamics over a time span of ~4 ps. The integration of Newton’s equations of motion was conducted with a timestep of 0.5 fs. The improved version of the fewest switches algorithm introduced by Hammes-Shiffer and Tully [33,34] was used in the calculations of the non-adiabatic transition probabilities between the *S*_1_ and *S*_0_ states; the Tully type fewest switches surface hopping algorithm allows switches between the *S*_1_ and *S*_0_ states [32].

For NEWTON-X/TURBOMOLE 7.50 calculations with the TD-DFT method, NVE-MDSH simulations were based on the overlap of the wavefunctions [31,32]. The populations of states, structures of the VdW and H-bond complexes, and dynamics before and after the non-radiative *S*_1_→*S*_0_ relaxation (surface hopping) were analysed in detail using the tools provided with NEWTON-X. The focus was on the reaction conditions (e.g. the exothermic *S*_1_→*S*_0_ relaxation temperatures and vertical excitation energies) and probabilities for formation of Gly and side products in the *S*_0_ state and interplay between the photo and thermal energies.

## 3. Results and discussion

### 3.1. Equilibrium structures

The equilibrium structures of molecules, VdW and H-bond precursors obtained from the DFT and CASPT2 methods are summarized in electronic supplementary material, table S1. It appears that all the equilibrium structures obtained from the DFT method are in excellent agreement with the CASPT2 optimized geometries. For example, two equilibrium structures are obtained for HOCOH, namely, the s- and m-forms, [HOCOH]^s,eq^ and [HOCOH]^m,eq^, respectively. The VdW and H-bond complexes formed from H_2_O and CO are suggested by the DFT method to be the global and local minimum energy geometries, [H_2_O…CO]^eq^ and [H_2_O…OC]^eq^, respectively. The equilibrium structures in electronic supplementary material, table S1, were used in the NEB reaction pathway optimizations.

### 3.2. The *S*_1_ and *S*_0_ potential energy curves

**Figure 3.**
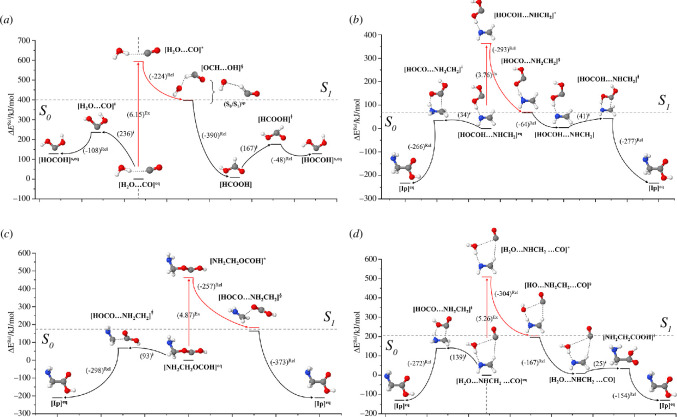
Examples of potential energy profiles for the formation of intermediates and Gly on the photochemical and *S*_0_ state pathways obtained from the TD-DFT, DFT and NEB methods. (*a*) Formation of [HOCOH]^s,eq^ from [H_2_O…CO]^eq^ (Ch (1)_Step (1)). (*b*) Formation of [Ip]^eq^ from [HOCOH...NHCH_2_]^eq^ (Ch (1)_Step (2)). (*c*) Formation of [Ip]^eq^ from [NH_2_CH_2_OCOH]^eq^ (Ch (3)_Step (2)). (*d*) Formation of [Ip]^eq^ from [H_2_O…NHCH_2_…CO]^eq^ (Ch (4)). The energies are in kJ mol^−1^ unless specified otherwise. Δ*E* ^Rel^ = relative energy with respect to the total energy of the binary complex precursor in the *S*_0_ state; (...)^Rel^ = relative energy with respect to the transition structure; (...)^ǂ^ = energy barrier; […]^*^ = *S*_0_→*S*_1_ vertically excited structure; […]^§^ = structure at the *S*_0_/*S*_1_ intersection; (...)^Ex^ = vertical excitation energy obtained from the TD-DFT method.

All the *S*_0_ and *S*_1_ potential energy curves and profiles obtained from the DFT, TD-DFT, CASPT2 and NEB methods are included in the electronic supplementary material. Examples of the potential energy profiles used in the discussion are illustrated in figure 3.

It appears that all the *S*_1_ potential energy curves obtained from the TD-DFT and NEB methods reveal barrierless energies for non-radiative *S*_1_→*S*_0_ relaxations of the vertically excited VdW and H-bond precursors and SA-CPMCSCF/6-31G(d) calculations confirm all the structures at the *S*_0_/*S*_1_ intersections (electronic supplementary material, table S2); for example, [H_2_O…CO]^*^→[OCH…OH]^§^ and [HOCOH...NHCH_2_]^*^→[HOCO…NH_2_CH_2_]^§^ in figure 3*a*,*b*, respectively.

It should be noted that because the CASPT2 potential energy curves were constructed using the geometries obtained from the TD-DFT and NEB methods, low energy barriers were observed on the *S*_1_ potential energy curves for [H_2_O…OC]^*^→[COH…OH]^§^ and [H_2_O…CO]^*^→[OCH…OH]^§^. To confirm barrierless potentials in the *S*_1_ state, all the degrees of freedom in the precursors, except the O–H…O H-bond and O…C VdW distances, were allowed to relax in CASPT2 calculations. The barrierless potentials obtained from the CASPT2 relax-scan calculations (electronic supplementary material, figures S2*a* and S3*a*) lead to the conclusion that the *S*_0_→*S*_1_ vertically excited H-bond and VdW precursors are instantly transformed into structures at the *S*_0_/*S*_1_ intersections, and the kinetic and thermodynamic properties of the photochemical pathways should be studied after non-radiative *S*_1_→*S*_0_ relaxations.

### 3.3. Bimolecular reactions

#### 3.3.1. Ch (1)_Step (1)

For the bimolecular reaction in Ch (1)_Step (1) (figure 1*b*), both [H_2_O…CO]^eq^ and [H_2_O…OC]^eq^ were used as the precursors in NEB calculations. The *S*_0_ potential energy profiles after the non-radiative *S*_1_→*S*_0_ relaxation show that the *S*_0_→*S*_1_ vertically excited [H_2_O…CO]^eq^ VdW precursor involves a higher energy barrier for the formation of [HOCOH]^s,eq^ than the [H_2_O…OC]^eq^ H-bond precursor; for [OCH…OH]^§^→[HOCOH]^s,eq^, Δ*E* ^ǂ^ = 167 kJ mol^−1^ (figure 3*a*), whereas Δ*E* ^ǂ^ = 123 kJ mol^−1^ for [COH…OH]^§^→[HOCOH]^s,eq^ (electronic supplementary material, figure S2*b*). The higher energy barrier could be attributed to the formation of [HCOOH] on a barrierless potential prior to the formation of [HOCOH]^s,eq^.

Similar results were obtained for the formation of [HOCOH]^m,eq^, for which the *S*_0_→*S*_1_ vertically excited [H_2_O…CO]^eq^ involves a high energy barrier due to the formation of HCOOH; for [OCH…OH]^§^→[HOCOH]^m,eq^, Δ*E* ^ǂ^ = 148 kJ mol^−1^ (electronic supplementary material, figure S3c). It should be noted that although [COH…OH]^§^→[HOCOH]^m,eq^ possesses a lower energy barrier (Δ*E* ^ǂ^ = 124 kJ mol^−1^ in electronic supplementary material, figure S2c), the formation of [HOCOH]^m,eq^ on this reaction pathway has to pass through [HOCOH]^s^ and the energy barrier for the interconversion between the s- and m-forms on the *S*_0_ state pathway is moderate, Δ*E* ^ǂ^ = 68 kJ mol^−1^ (electronic supplementary material, figure S3*d*). Therefore, only [HOCOH]^s,eq^ was chosen in further study on Ch (1). Comparison of the above-discussed photochemical pathways with the *S*_0_ state pathways shows considerably lower energy barriers; for [H_2_O…CO]^eq^→[HOCOH]^s,eq^ and [H_2_O…OC]^eq^→[HOCOH]^s,eq^ on the *S*_0_ state pathways, Δ*E* **^ǂ^** = 236 (figure 3*a*) and 242 kJ mol^−1^ (electronic supplementary material, figure S2*c*), respectively. These high energy barriers result mainly from the O−H→O unimolecular isomerization on the *S*_0_ state pathways.

#### 3.3.2. Ch (2)_Step (1)

Because H_2_ and CO_2_ are stable molecules, the formation of [HOCOH]^eq^ on both photochemical and *S*_0_ state pathways involves considerably high energy barriers, Δ*E* ^ǂ^ > 230 kJ mol^−1^. Therefore, irradiation seems not to help promote the formation of [HOCOH]^eq^ in Ch (2)_Step (1). It should be mentioned that the potential energy profile reveals the formation of [HCOOH]^eq^ from [HCOO…H]^§^ on a barrierless potential (electronic supplementary material, figure S4*b*). Because [HCOOH]^eq^ could be generated as a side product from [H_2_O…CO]^eq^ and [H_2_…CO_2_]^eq^, and [HCOOH]^eq^ is also a precursor in Ch (3)_Step (1), an attempt was made to study the possibility for the formation of [HOCOH]^eq^ from [HCOOH]^eq^. The results (electronic supplementary material, figure S5*a*) show that the *S*_0_→*S*_1_ vertically excited [HCOOH]^eq^ could non-radiatively relax to the *S*_0_ state, and after the non-radiative *S*_1_→*S*_0_ relaxation, [OCH…OH]^§^→[HOCOH]^eq^ involves a moderate energy barrier (Δ*E* ^ǂ^ = 123 kJ mol^−1^). Therefore, [HCOOH]^eq^ could be an effective precursor for the formation of [HOCOH]^eq^.

#### 3.3.3. Ch (1)_Step (2)

The potential energy profiles for the formation of Gly ([Ip]^eq^) from [HOCOH−NHCH_2_]^eq^ are shown in figure 3*b*. It appears that the energy barriers for the formation of [Ip]^eq^ on the photochemical and *S*_0_ state pathways are not significantly different. The *S*_0_→*S*_1_ vertically excited H-bond precursor barrierlessly relaxes to the *S*_0_/*S*_1_ intersection, and [HOCO…NH_2_CH_2_]^§^→[Ip]^eq^ in the *S*_0_ state has a low energy barrier, Δ*E* ^ǂ^ = 41 kJ mol^−1^; the energy barrier for [HOCOH−NHCH_2_]^eq^→[Ip]^eq^ on the *S*_0_ state pathway is slightly lower, Δ*E* ^ǂ^ = 34 kJ mol^−1^. These results lead to the conclusion that the formation of Gly in Ch (1)_Step (2) could occur on both photochemical and *S*_0_ state pathways.

#### 3.3.4. Ch (3)_Step (1) and Step (2)

For the bimolecular reactions in Ch (3), the potential energy profiles reveal that although the formation of [Ip]^eq^ from [HOCO…NH_2_CH_2_]^§^ (Ch (3)_Step (2) in figure 3*c*) involves a barrierless energy, the formation of [NH_2_CH_2_OCOH]^eq^ on the photochemical pathway (Ch (3)_Step (1) in electronic supplementary material, figure S6*a*) possesses a moderate energy barrier in the *S*_0_ state (Δ*E* ^ǂ^ = 117 kJ mol^−1^), whereas the energy barrier for the formation of [Ip]^eq^ directly from [HCOOH…NHCH_2_]^eq^ on the *S*_0_ state pathway has a comparable energy barrier (Δ*E* ^ǂ^ = 119 kJ mol^−1^; electronic supplementary material, figure S6*c*). These results lead to the conclusion that the photochemical process does not help promote the formation of [Ip]^eq^ in Ch (3) and [HCOOH…NHCH_2_]^eq^→[Ip]^eq^ could occur on the *S*_0_ state pathway.

Based on the above discussion, one can conclude that for bimolecular reactions, the photochemical pathways in Ch (1) and (3) are more favourable than those in Ch (2). For the photochemical pathways in Ch (1), the non-radiative *S*_1_→*S*_0_ relaxation of the vertically excited H_2_O…CO VdW precursor could generate HCOOH as a side product, from which HOCOH could be formed. In addition, the formation of Gly from the HCOOH−CH_2_NH H-bond complex in Ch (1)_Step (2) is energetically feasible on both photochemical and *S*_0_ state pathways.

### 3.4. Termolecular reactions

For the termolecular reactions in Ch (4), the potential energy profiles in figure 3*d* show that the *S*_0_→*S*_1_ vertically excited [H_2_O…NHCH_2_…CO]^eq^ precursor instantly relaxes to the *S*_0_/*S*_1_ intersection, and after the non-radiative *S*_1_→*S*_0_ relaxation, [HO…NH_2_CH_2_…CO]^§^→[Ip]^eq^ proceeds on a low barrier potential (Δ*E* ^ǂ^ = 25 kJ mol^−1^), whereas [H_2_O…NHCH_2_…CO]^eq^→[Ip]^eq^ on the *S*_0_ state pathway involves the binary complex [HOCO…NH_2_CH_2_]^ǂ^ transition structure with a moderate energy barrier (Δ*E* **^ǂ^** = 139 kJ mol^−1^).

It should be noted, for example, that although the structure after the *S*_1_→*S*_0_ relaxation ([H_2_O…NHCH_2_…CO] in figure 3*d*) and reaction precursor are similar, they are not exactly the same. Based on different NEB gradients, the photochemical pathway (right-hand side) and *S*_0_ state pathway (left-hand side) proceed through different transition states; on the photochemical pathway, the *S*_0_/*S*_1_ structure ([HO…NH_2_CH_2_…CO]^§^) is the starting geometry in the NEB calculations, whereas on the *S*_0_ state pathway, the equilibrium structure ([H_2_O…NHCH_2_…CO]^eq^) is the starting geometry. Although the photochemical pathway possesses lower Δ*E* ^ǂ^, without the effect of thermal energy, it is difficult to decide on which pathway the reaction will proceed. Therefore, to complete the mechanistic study, TST calculations and NVE-MDSH simulations must be performed to explore favourable thermodynamic conditions, time evolutions and statistics of the formation of the intermediates and products on the candidate reaction pathways.

For the termolecular reaction in Ch (5), while the reaction on the *S*_0_ state pathway is similar to that in Ch (4) (Δ*E* ^ǂ^ = 139 kJ mol^−1^), the vertically excited [H_2_…NHCH_2_…CO_2_]^eq^ precursor readily transforms into [HOCO…NH_2_CH_2_]^§^, and after the non-radiative *S*_1_→*S*_0_ relaxation, the breaking of the C−O covalent bond and N−H→Ο proton/H-atom transfer lead to [H_2_O…NHCH_2_…CO]^eq^ (electronic supplementary material, figure S7*b*), which is the precursor in Ch (4). These findings suggest that the photochemical process in Ch (5) does not help promote the formation of [Ip]^eq^. The photochemical pathways obtained based on the DFT, TD-DFT and NEB methods will be regarded as static reaction pathways.

### 3.5. Kinetic and thermodynamic properties

To further study the candidate photochemical pathways (Ch (1), (3) and (4)), kinetic and thermodynamic properties were computed using the TST method, from which all the results are summarized in electronic supplementary material, tables S3−S5. Outstanding results used in the discussion are included in electronic supplementary material, tables S6−S8. Because NVE-MDSH simulations (to be discussed in the forthcoming subsections) confirm no stable intermediate in the *S*_1_ state, the discussions are focused only on the scenarios after the non-radiative *S*_1_→*S*_0_ relaxations. In addition, because the NVE-MDSH results revealed that the average exothermic *S*_1_→*S*_0_ relaxation temperatures for all the photochemical pathways are high, the discussion will emphasize the thermodynamic spontaneity for the formation of Gly and side products.

#### 3.5.1. Quantum mechanical effect

Because the average exothermic *S*_1_→*S*_0_ relaxation temperatures obtained from NVE-MDSH simulations are higher than *T*_c_, the quantum mechanical effect is concluded not to be important in the present systems; the highest quantum mechanical effect is for [HOCOH−NHCH_2_]→[HOCO−CH_2_NH_2_]^ǂ^, *T*_c_ = 216 K (Ch (1)_Step (2) in electronic supplementary material, table S6*b*), below which the rate constants for the O−H→N proton/H-atom transfer are significantly different, e.g. in electronic supplementary material, table S3*e*, at *T* = 100 K, ${\text{k}}_{\text{f}}^{\text{Class}}$ = 2.64 × 10^−10^, ${\text{k}}_{\text{f}}^{\text{Q-vib}}$ = 2.97 × 10^−6^ and ${\text{k}}_{\text{f}}^{\text{S-Wig}}$ = 2.59 × 10^−5^ s^−1^, whereas at high temperatures, e.g. at *T* = 1500 K, ${\text{k}}_{\text{f}}^{\text{Q-vib}}$ and ${\text{k}}_{\text{f}}^{\text{S-Wig}}$ are comparable, 4.20 × 10^10^ and 4.34 × 10^10^ s^−1^, respectively. Therefore, it is reasonable to use only *k* ^Q-vib^ in further analysis on the exothermic *S*_*1*_→*S*_0_ relaxation processes.

#### 3.5.2. Thermodynamic spontaneity

For the photochemical process in Ch (1)_Step (1), the analysis of the TST results suggests that after the exothermic *S*_1_→*S*_0_ relaxation, [OCH…OH]^§^→[HOCOH]^s,eq^ is thermodynamically more favourable at low temperature, for example, at 1200 K, $\Delta G^{˚,{\left(I\right)}^{§}\to (IV)}$ = −201 kJ mol^−1^, whereas at 4500 K, $\Delta G^{˚,{\left(I\right)}^{§}\to (IV)}$ = −79 kJ mol^−1^, with ${\text{k}}_{\text{f}}^{\text{Q-vib}}$ = 1.46 × 10^2^ and 2.14 × 10^7^ s^−1^ (electronic supplementary material, table S6*a*), respectively. The plot of the free energy barriers ( ${\Delta G}_{r}^{ǂ,(I{)}^{§}\leftarrow \left(II\right)}$ and ${\Delta G}_{f}^{ǂ,(II)\to (III)ǂ}$ ) versus temperature suggests the spontaneous temperature for the formation of [HOCOH]^ǂ^ in Ch (1)_Step (1) below *T*_s_ = 6321 K (electronic supplementary material, figure S8*a*). The TST results suggest that at the same temperatures, Ch (1)_Step (2) is both kinetically and thermodynamically more favourable than Ch (1)_Step (1), $\Delta G^{˚,{\left(I\right)}^{§}\to (IV)}$ = −260 and −221 kJ mol^−1^, with ${\text{k}}_{\text{f}}^{\text{Q-vib}}$ = 2.07 × 10^10^ and 3.09 × 10^11^ s^−1^, respectively, and the formation of Gly (structure Ip) could be spontaneous below *T*_s_ = 974 K in electronic supplementary material, table S6*b*.

For the photochemical pathways in Ch (3), the lowest spontaneous temperature is for Ch (3)_Step (1), *T*_s_ = 634 K for [HOCO…NH_2_CH_2_]^§^→[HCOOH…NHCH_2_]^ǂ^, and at 4500 K, $\Delta G^{˚,{\left(I\right)}^{§}\to (IV)}$ = 701 kJ mol^−1^, with ${\text{k}}_{\text{f}}^{\text{Q-vib}}$ = 4.61 × 10^7^ s^−1^ (electronic supplementary material, table S7*a*). Because the exothermic *S*_1_→*S*_0_ relaxation temperatures are generally higher than this *T*_s_, the consecutive reaction pathway in Ch (3)_Step (1) is concluded not to be thermodynamically favourable. For Ch (3)_Step_2, the vertically excited structure [NH_2_CH_2_OCOH]^eq^ readily relaxes to the *S*_0_/*S*_1_ intersection, and the formation of Gly ([Ip]^eq^) is on a kinetically and thermodynamically favourable reaction pathway; electronic supplementary material, table S7*b* shows that for [HOCO…NH_2_CH_2_]^§^→[Ip]^eq^, $\Delta G^{˚,{\left(I\right)}^{§}\to (II)}$ = −33 kJ mol^−1^ at *T* = 3115 K. Based on these analyses, Ch (3) could also be a candidate photochemical pathway for the formation of Gly.

For the termolecular reaction in Ch (4), due to the low energy barrier (Δ*E* **^ǂ^** = 25 kJ mol^−1^) after the exothermic *S*_1_→*S*_0_ relaxation, the formation of Gly is thermodynamically favourable, e.g. at 4500 K, $\Delta G^{˚,{\left(I\right)}^{§}\to (IV)}$ = −181 kJ mol^−1^ and ${\text{k}}_{\text{f}}^{\text{Q-vib}}$ = 4.52 × 10^4^ s^−1^ (electronic supplementary material, table S8). The spontaneous temperature for the formation of the [NH_2_CH_2_COOH]^ǂ^ transition structure is *T*_s_ = 3763 K. It should be noted that although the termolecular reaction in Ch (4) possesses a low energy barrier, and is thermodynamically favourable, the high exothermic *S*_1_→*S*_0_ relaxation energy could lead to separation of the weakly associated H_2_O…CH_2_NH…CO ternary complex into single molecules. This issue will be discussed based on the NVE-MDSH results.

### 3.6. Dynamic reaction pathways

The dynamics of the photochemical pathways in Ch (1), (3) and (4) are discussed in detail using the NVE-MDSH results. The emphases are on the statistics and effect of the exothermic *S*_1_→*S*_0_ relaxation temperature on the thermodynamic spontaneity, as well as the thermal and photo selectivity. Because the reaction pathways after the non-radiative *S*_1_→*S*_0_ relaxations are thermally selective [13], they can be classified into two categories, as in the case of the Gibbs free energy profiles (figure 2). For the single-step reaction pathway, intermediate(s) or product(s) could be formed directly from the structure at the *S*_0_/*S*_1_ intersection ((I)^§^→(II)), characterized by an average exothermic *S*_1_→*S*_0_ relaxation temperature. However, more than one average exothermic *S*_1_→*S*_0_ relaxation temperature corresponding to the formation of transient intermediate(s) and product(s) is considered characteristic of consecutive reaction pathways, (I)^§^→(II) followed by (II)⇌(III)^ǂ^→(IV).

#### 3.6.1. Pathway statistical analysis

Starting from 307 Wigner-sampled VdW and H-bond precursors in Ch (1), (3) and (4), 62 NVE-MDSH simulations are without *S*_1_→*S*_0_ surface hopping. Therefore, 245 NVE-MDSH simulations are used in the dynamic analysis (100%). It appears that in cold interstellar media (NVE ensemble), the formation of Gly is feasible in photochemical pathways with several side products, depending upon the Δ*E* ^Ex^ of the precursors, exothermic *S*_1_→*S*_0_ relaxation and spontaneous temperatures. For example, the HOCOH intermediate in Ch (1) (figure 3*a*) could be formed from the HCOOH precursor (1.63% in electronic supplementary material, table S9*a*), and Gly could be generated in Ch (1)_Step (2) (0.82% in electronic supplementary material, table S9*b*). It also appears that 11 side products, such as OH, HCN, HNC, HCOOH and H_2_ (electronic supplementary material, table S10), are observed in the NVE-MDSH simulations.

**Figure 4.**
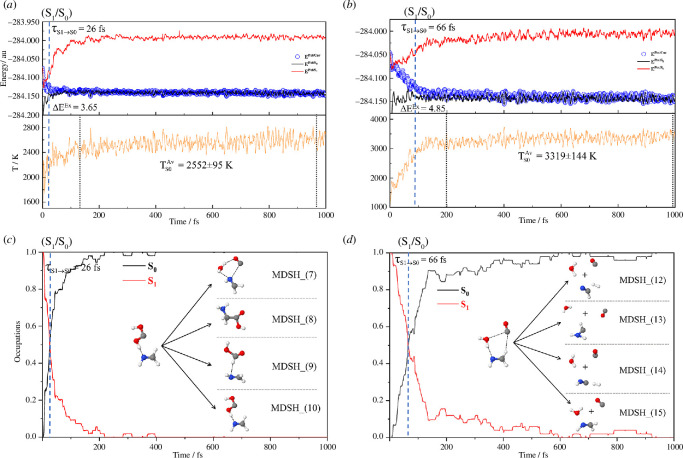
(*a*,*c*) The total average results obtained for Ch (1)_Step (2) (MDSH_(7)−(10), *n*_*T*_ = 53). (*b*,*d*) The total average results obtained for Ch (4) (MDSH_(12)−(15), *n*_*T*_ = 51). Temperature and simulation time are in K and fs, respectively. Δ*E* ^Ex^ = *S*_0_→*S*_1_ vertical excitation energy; MDSH_(*n*) = single-step/consecutive reaction path *n*; ${\text{T}}_{\text{S0}}^{\text{Av}}$ = average temperature with standard deviation (s.d.); ${\text{τ}}_{{\text{S}}_{\text{1}}\text{→}{\text{S}}_{\text{0}}}$ = non-radiative *S*_1_→*S*_0_ relaxation time.

To explain these findings, the NVE-MDSH results on the VdW and H-bond precursors are systematically analysed. Because the total average exothermic *S*_1_→*S*_0_ relaxation temperatures ( ${\left\langle \text{T}\right\rangle }_{\text{S0}}^{\text{Ch(n)}}$ , *n* = 1, 3 and 4) for the proposed photochemical pathways are not well separated and cannot be used to differentiate (I)^§^→(II) and (I)^§^→(II)⇌(III)^ǂ^→(IV) (e.g. figure 4*a*,*b* for Ch (1)_Step (2) and Ch (4), respectively), to study the characteristic temperatures for the formation of transient intermediates and products, the 245 NVE-MDSH results are subdivided into 15 dynamic reaction pathways based on the precursors, intermediates and products.

**Figure 5.**
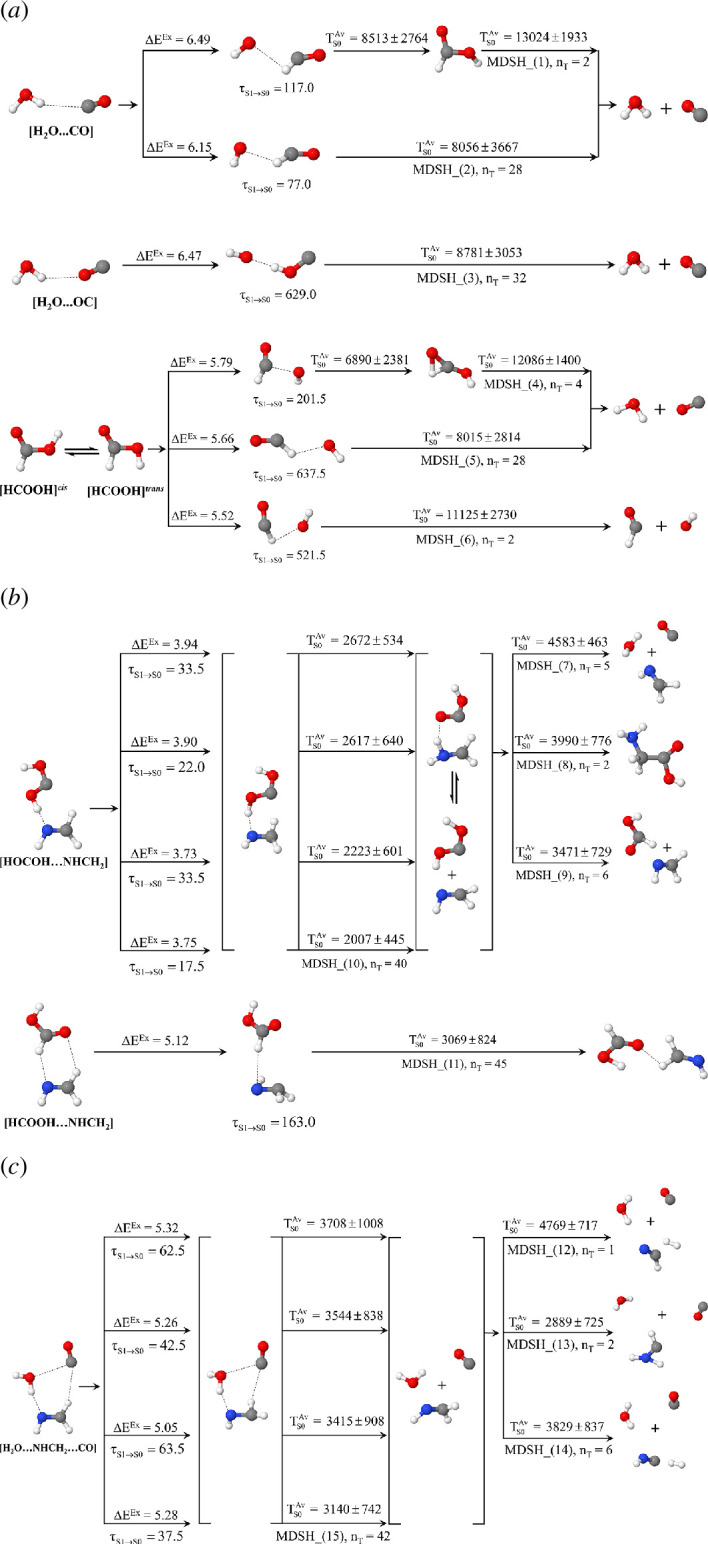
Representative reaction pathways after the *S*_1_→*S*_0_ relaxation obtained from NVE-MDSH simulations. Temperature and simulation time are in K and fs, respectively. (*a*) MDSH_(1)−(6). (*b*) MDSH_(7)−(11). (*c*) MDSH_(12)−(15). Δ*E* ^Ex^ = *S*_0_→*S*_1_ vertical excitation energy (eV); MDSH_(*n*) = dynamic reaction pathway (*n*); *n*_*T*_ = number of MDSH_(*n*) observed; ${\text{T}}_{\text{S0}}^{\text{Av}}$ = average temperature with s.d.; ${\text{τ}}_{{\text{S}}_{\text{1}}\text{→}{\text{S}}_{\text{0}}}$ = *S*_1_→*S*_0_ relaxation time.

The representative dynamic reaction pathways are selected and included in figure 5, in which MDSH_(1)−(3) and (7)−(10) are for the bimolecular reactions in Ch (1)_Step (1) and Ch (1)_Step (2), respectively. MDSH_(11) is the representative NVE-MDSH results on the bimolecular reaction in Ch (3)_Step_(1), whereas MDSH_(12)−(15) are for the termolecular reaction in Ch (4). The NVE-MDSH results on the *S*_0_→*S*_1_ vertically excited HCOOH are MDSH_(4)−(6), in which the formation of the HOCOH transient intermediate is in MDSH_(4).

Because NVE-MDSH simulations on Ch (3)_Step_(1) (18.4% in figure 5*b*) do not generate the NH_2_CH_2_OCOH intermediate because of the high exothermic *S*_1_→*S*_0_ relaxation temperature (MDSH_(11)), the photochemical pathway in Ch (3) might not be feasible in cold interstellar media and therefore was ruled out from the discussion.

**Figure 6.**
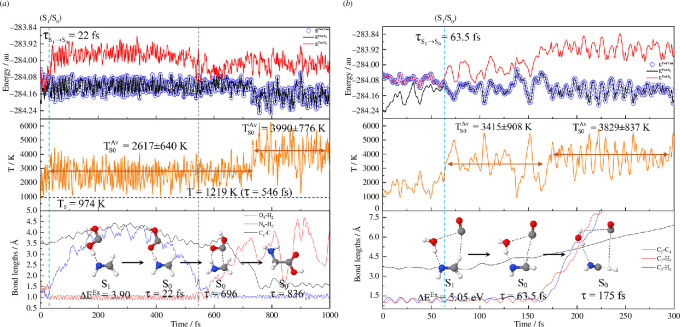
Examples of the results on the representative reaction pathways after the *S*_1_→*S*_0_ relaxation obtained from NVE-MDSH simulations. (*a*) MDSH_(8) obtained using [HOCOH… CH_2_NH] as the precursor. (*b*) MDSH_(14) obtained using [H_2_O…NHCH_2_…CO] as the precursor. *E* ^Pot/S0^, *E* ^Pot/S1^ and *E* ^Pot/Cur^ = potential energies in the *S*_0_, *S*_1_ and current states.

The time evolutions of the structures, energetics and exothermic *S*_1_→*S*_0_ relaxation temperatures for the formation of Gly from the bimolecular reaction in Ch (1)_Step (2) (MDSH_(8)) and side products from the termolecular reaction in Ch (4) (MDSH_(14)) are included in figure 6 as an example.

#### 3.6.2. Bimolecular reaction

The NVE-MDSH results on Ch (1)_Step (1) reveal that all the *S*_0_→*S*_1_ vertically excited Wigner-sampled H_2_O…CO VdW and H_2_O−OC H-bond precursors (25.3%, ${\text{n}}_{\text{T}}^{\text{Ch(1)\_Step(1)}}$ = 62 in electronic supplementary material, table S9*a*) readily relax to the *S*_0_ state with the total average *S*_1_→*S*_0_ relaxation time, ${\left\langle \tau \right\rangle }_{S1\to S0}^{Ch(1)\_Step(1)}$ = 189 fs (electronic supplementary material, figure S9*a*). For MDSH_(2) and (3), the proton/H-atom exchanges in the H_2_O…CO VdW and H_2_O−OC H-bond complexes are on single-step reaction pathways, and the *S*_0_/*S*_1_ structures relax to the precursors (figure 5*a*), ${\text{T}}_{\text{S0}}^{\text{Av, MDSH\_(2)}}$ = 8056 ± 3667 and ${\text{T}}_{\text{S0}}^{\text{Av, MDSH\_(3)}}$ = 8781 ± 3053 K. It appears that after the non-radiative *S*_1_→*S*_0_ relaxations, HOCOH cannot be formed from the H_2_O…CO VdW and H_2_O−OC H-bond precursors because ${\text{T}}_{\text{S0}}^{\text{Av, MDSH\_(1)-(3)}}$ are considerably higher than the spontaneous temperature for the formation of HOCOH, *T*_s_ = 6321 K (electronic supplementary material, table S6*a*), implying the non-synergistic effect of the photochemical and thermochemical pathways. In other words, the exothermic *S*_1_→*S*_0_ relaxation process oversupplies the thermal energy for the formation of HOCOH.

Because MDSH_(1) in figure 5*a* reveals HCOOH as a transient intermediate (0.8%, ${\text{n}}_{\text{T}}^{\text{MDSH\_(1)}}$ = 2) at ${\text{T}}_{\text{S0}}^{\text{Av, MDSH\_(1)}}$ = 8513 ± 2764 K (τ < 135 fs), NVE-MDSH simulations on the *S*_0_→*S*_1_ vertically excited Wigner-sampled HCOOH precursors were performed. MDSH_(4)−(6) in figure 5*a* show that C−OH covalent bond cleavage followed by C−H→O proton/H-atom transfer dominates, leading to CO and H_2_O as the major products (13.1%, ${\text{n}}_{\text{T}}^{\text{MDSH\_(4)-(5)}}$ = 32). Whereas HOCOH is formed only as a transient intermediate (1.6%, ${\text{n}}_{\text{T}}^{\text{MDSH\_(4)}}$ = 4), because ${\text{T}}_{\text{S0}}^{\text{Av, MDSH\_(4)}}$ = 6890 ± 2381 K is higher than *T*_s_ (6321 K in electronic supplementary material, table S6*a*), there is a non-synergistic effect of thermal energy in the consecutive reaction pathways.

Starting from the *S*_0_→*S*_1_ vertically excited Wigner-sampled HOCOH−CH_2_NH H-bond precursors in Ch (1)_Step (2), NVE-MDSH simulations show that the total average non-radiative *S*_1_→*S*_0_ relaxation time is very short, ${\left\langle \text{τ}\right\rangle }_{\text{S1→S0}}^{\text{Ch(1)\_Step(2)}}$ = 26 fs (figure 4*a*), and after the non-radiative *S*_1_→*S*_0_ relaxation, Gly is formed in MDSH_(8) (0.8%, ${\text{n}}_{\text{T}}^{\text{MDSH\_(8)}}$ = 2) with three side products (HCOOH, CO and H_2_O) generated in MDSH_(7) and (9) (4.5%, ${\text{n}}_{\text{T}}^{\text{MDSH\_(7),(9)}}$ = 11). Figure 5*b* and electronic supplementary material, table S9*b*, show that MDSH_(7)−(9) are on consecutive reaction pathways and proton/H-atom shuttling in the N−H…O H-bond represents the characteristic dynamics at the lowest temperature; in MDSH_(10) (16.3%, ${\text{n}}_{\text{T}}^{\text{MDSH\_(10)}}$ = 40), ${\text{T}}_{\text{S0}}^{\text{Av,MDSH\_(10)}}$ = 2007 ±445 K and the N−H→O proton/H-atom transfer brings the *S*_0_/*S*_1_ structure back to the H-bond precursors.

The NVE-MDSH results in figure 5*b* also show that above ${\text{T}}_{\text{S0}}^{\text{Av,MDSH\_(10)}}$ , the formation of molecular products is thermally selective. The N−H→C proton/H-atom transfer in MDSH_(9) (2.4%, ${\text{n}}_{\text{T}}^{\text{MDSH\_(9)}}$ = 6) at ${\text{T}}_{\text{S0}}^{\text{Av, MDSH\_(9)}}$ = 2223 ± 601 K generates HCOOH, whereas the N−H→O proton/H-atom transfer in MDSH_(7) (2.0%, ${\text{n}}_{\text{T}}^{\text{MDSH\_(7)}}$ = 5) is at ${\text{T}}_{\text{S0}}^{\text{Av, MDSH\_(7)}}$ = 2672 ± 534 K, and C−OH bond breaking and formation of H_2_O and CO take place at the highest average temperature, ${\text{T}}_{\text{S0}}^{\text{Av, MDSH\_(7)}}$ = 4583 ± 463 K.

For Gly formation, MDSH_(8) (0.8%, ${\text{n}}_{\text{T}}^{\text{MDSH\_(8)}}$ = 2) in figure 5*b* shows that the N−H→O proton/H-atom exchange is at ${\text{T}}_{\text{S0}}^{\text{Av, MDSH\_(8)}}$ = 2617 ± 640 K, and the average temperature increases after C−C covalent bond formation, ${\text{T}}_{\text{S0}}^{\text{Av, MDSH\_(8)}}$ = 3990 ± 776 K. The low percentage for Gly formation could be attributed again to high ${\text{T}}_{\text{S0}}^{\text{Av, MDSH\_(8)}}$ compared with *T*_s_ (electronic supplementary material, table S6*b*); the Gibbs free energy plots in electronic supplementary material, figure S8*d*, suggest *T*_s_ = 974 K, whereas the formation of Gly in MDSH_(8) begins at a higher temperature, *T* = 1219 K at 546 fs (figure 6*a*).

#### 3.6.3. Termolecular reactions

The NVE-MDSH results on the *S*_0_→*S*_1_ vertically excited Wigner-sampled H_2_O…CH_2_NH…CO precursors show that although the termolecular reaction in Ch (4) is suggested by the TST method to be strongly spontaneous below *T*_s_ = 3763 K (e.g. at *T* = 3115 K, $\Delta G^{˚,{\left(I\right)}^{§}\to (II)}$ = −205 kJ mol^−1^ in electronic supplementary material, table S8), due to the high exothermic *S*_1_→*S*_0_ relaxation temperature, Gly is unlikely to be formed on this photochemical pathway, especially in extremely low-density interstellar media. The separation of the weakly associated H_2_O…CH_2_NH…CO ternary complexes into single molecules at ${\text{T}}_{\text{S0}}^{\text{Av, MDSH\_(15)}}$ = 3140 ± 742 K (figure 5*c*) is the major product in MDSH_(15) (17.1%, ${\text{n}}_{\text{T}}^{\text{MDSH\_(15)}}$ = 42). However, the NVE-MDSH results on Ch (4) show several molecular products resulting mainly from covalent bond dissociation/formation and isomerization in CH_2_NH (3.7%, ${\text{n}}_{\text{T}}^{\text{MDSH\_(12)-(14)}}$ = 9); H_2_ and HCN in MDSH_(12) (0.4%, ${\text{n}}_{\text{T}}^{\text{MDSH\_(12)}}$ = 1) at ${\text{T}}_{\text{S0}}^{\text{Av, MDSH\_(12)}}$ = 4769 ± 717 K; CHNH_2_ (amino methylene) in MDSH_(13) (0.8%, ${\text{n}}_{\text{T}}^{\text{MDSH\_(13)}}$ = 2) at ${\text{T}}_{\text{S0}}^{\text{Av, MDSH\_(13)}}$ = 2889 ± 725 K; and H_2_ and HNC in MDSH_(14) (2.4%, ${\text{n}}_{\text{T}}^{\text{MDSH\_(14)}}$ = 6) at ${\text{T}}_{\text{S0}}^{\text{Av, MDSH\_(14)}}$ = 3829 ± 837 K (figure 6*b*).

#### 3.6.4. Photoselectivity of precursors

**Figure 7.**
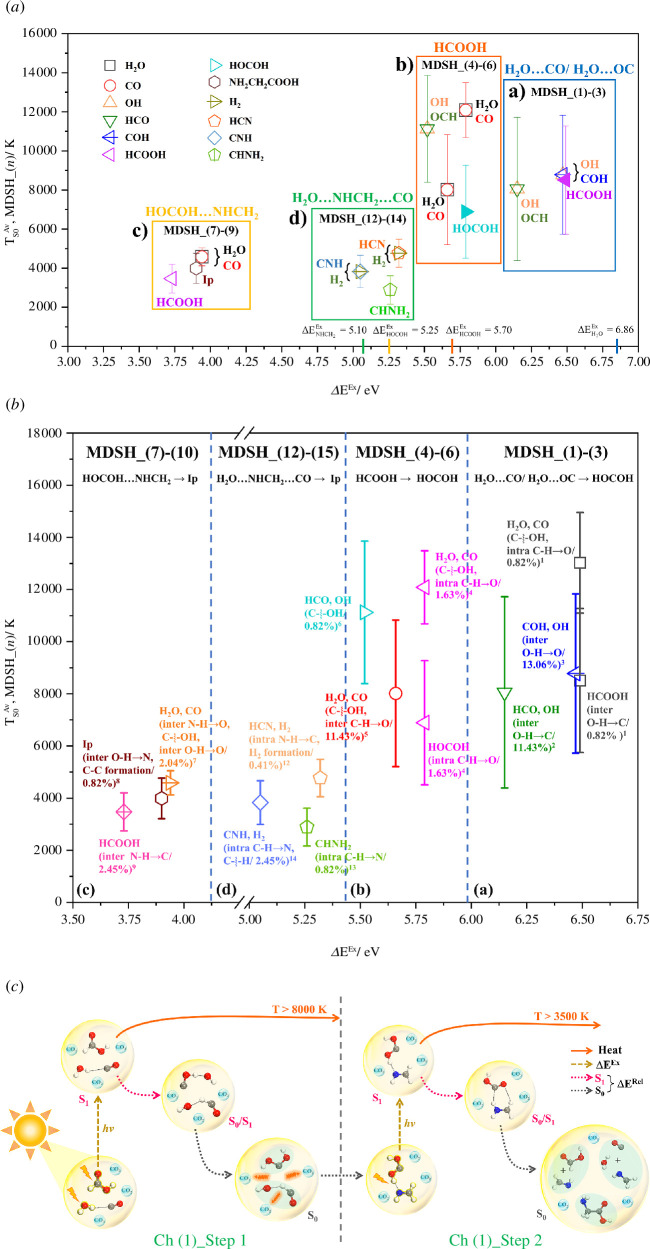
(*a*) Correlations between the average exothermic *S*_1_→*S*_0_ relaxation temperatures ( ${\text{T}}_{\text{S0}}^{\text{Av, MDSH\_(n)}}$ , *n* = 1−9 and 12−14) and *S*_0_→*S*_1_ vertical excitation energies (Δ*E* ^Ex^) for the representative MDSH_(*n*). (*b*) Photothermal correlation matrix showing the temperatures at which molecular processes and intermediates/products occur. (*c*) A proposed strategy for formation of Gly in heat-absorbing gas CO_2_.

The correlations between the average exothermic *S*_1_→*S*_0_ relaxation temperatures and *S*_0_→*S*_1_ vertical excitation energies for the representative single-step and consecutive reaction pathways ( $T_{S0}^{Av,MDSH\_(n)}$ , *n* = 1−9 and 12−14, and Δ*E* ^Ex^ in figure 5) are shown in figure 7, together with the s.d. The statistics for the formation of the intermediate(s)/molecular product(s) through covalent bond breaking and/or formation, proton/H-atom exchange, and isomerization are summarized in electronic supplementary material, table S10. These pieces of information are used to discuss the thermal selectivity for the formation of molecular products.

The correlation diagram in figure 7*a* shows four domains labelled (a)−(d), in which (a) and (b) are for Ch (1)_Step (1) (MDSH_(1)−(6)) occurring at high ${\text{T}}_{\text{S0}}^{\text{Av, MDSH\_(1)-(6)}}$ with high s.d., whereas (c) and (d) are for Ch (1)_Step (2) (MDSH_(7)−(10)) and Ch (4) (MDSH_(12)−(14)) with lower ${\text{T}}_{\text{S0}}^{\text{Av, MDSH\_(7)-(14)}}$ and lower s.d., respectively. Analysis of the representative molecular products in domain (a) (MDSH_(1)−(3)) shows that because Δ*E* ^Ex^ of the Wigner-sampled precursors (Δ*E* ^Ex^ = 6.15−6.49 eV) are close to that of H_2_O (Δ*E* ^Ex^ = 6.86 eV in electronic supplementary material, table S1), the photoexcitation is unimolecular, occurring mainly at H_2_O and leads to proton/H-atom exchange between H_2_O and CO and eventually separated precursor molecules as the products (electronic supplementary material, figures S9*a* and S10*a)*.

Likewise, Δ*E* ^Ex^ in domain (d) (MDSH_(12)−(15) in figure 7*a*) suggests that the *S*_0_→*S*_1_ vertical excitation energies of the Wigner-sampled H_2_O…CH_2_NH…CO precursors are compatible with Δ*E* ^Ex^ of CH_2_NH, and the photochemical processes occur mainly at CH_2_NH, resulting in HCN, HNC and H_2_; the former are Δ*E* ^Ex^ = 5.05−5.32 eV, and the latter is Δ*E* ^Ex^ = 5.10 eV. In contrast, due to the strong O−H…N H-bond (Δ*E* = −50.1 kJ mol^−1^) and intermolecular donor–acceptor charge transfer (CT) in the HOCOH−CH_2_NH precursor, Δ*E* ^Ex^ in domain (c) (MDSH_(7)−(10)) is redshifted (~1.38 eV) from Δ*E* ^Ex^ of the isolated HOCOH and CH_2_NH.

#### 3.6.5. Thermal selectivity and molecular processes

To acquire fundamental information to control thermochemical processes, molecular mechanisms (processes) underlying the formation of molecular products after the exothermic *S*_1_→*S*_0_ relaxation in figure 7*a* are studied in detail. In this study, the Δ*E* ^Ex^ of the Wigner-sampled precursors and the characteristic temperatures for covalent bond dissociation and inter- and intramolecular proton/H-atom exchanges are analysed, classified and included in the photothermal correlation matrix in figure 7*b*. The analysis of figure 7*b* suggests that starting from the same type of precursor, the exothermic *S*_1_→*S*_0_ relaxation temperatures of consecutive reaction pathways are higher than those of single-step reaction pathways due to formation and/or dissociation of the transient intermediates.

It appears that for strong N−H…O H-bonds, the inter- and intramolecular proton/H-atom exchanges dominate in the low average exothermic *S*_1_→*S*_0_ relaxation temperature range and low Δ*E* ^Ex^ (domains (c) and (d) in figure 7*b*); ${\text{T}}_{\text{S0}}^{\text{Av,inter N-H→C}}$ < ${\text{T}}_{\text{S0}}^{\text{Av,inter O-H→N}}$ < ${\text{T}}_{\text{S0}}^{\text{Av,inter O-H→O}}$ (MDSH_(7)−(10)) and ${\text{T}}_{\text{S0}}^{\text{Av,intra C-H→N}}$ < ${\text{T}}_{\text{S0}}^{\text{Av,intra N-H→C}}$ (MDSH_(12)−(15)), respectively. However, the proton/H-atom exchanges in VdW and weak H-bonds dominate at high Δ*E* ^Ex^ and in the high average exothermic *S*_1_→*S*_0_ relaxation temperature range; in domain (a) in figure 7*b*, ${\text{T}}_{\text{S0}}^{\text{Av,inter O-H→C}}$ < ${\text{T}}_{\text{S0}}^{\text{Av,inter O-H→O}}$ < ${\text{T}}_{\text{S0}}^{\text{Av,intra C-H→O}}$ . The C−OH covalent bond cleavage ( $T_{S0}^{Av,C-§-OH}$ ) occurs in the highest average exothermic *S*_1_→*S*_0_ relaxation temperature range (domains (a) and (b) in figure 7*b*).

#### 3.6.6. Photo-to-thermal control strategies

Because the theoretical results in this work suggested that the formation of intermediates or products requires heat to overcome energy barriers in the consecutive reaction pathways, but the average exothermic *S*_1_→*S*_0_ relaxation temperatures are exceedingly higher than *T*_s_ (figure 7*b*), a photo-to-thermal control strategy is required in the experiment; the non-synergistic effect of the photochemical and thermochemical pathways could be one of the reasons for the lack of product or low product yields.

To enhance the product yields, a heat transfer or removal mechanism through inert gas molecules could be incorporated in the photochemical reaction chamber to carry away excess heat and cool down the temperature to below *T*_s_. For example, for the formation of the HOCOH intermediate and Gly in Ch (1), the reaction temperatures after the exothermic *S*_1_→*S*_0_ relaxation should be controlled below *T*_s_ = 6321 and 974 K, respectively. In this case, non-reactive heat transfer gases (diluent gases), such as He, Ar, N_2_, CO_2_ or H_2_, could be used as ‘heat absorbing gases’ [35], among which CO_2_ could be a reasonable choice due to its high heat transfer capability and high Δ*E* ^Ex^ compared with the other VdW and H-bond precursors (figure 7*c*).

Alternatively, because photochemical reactions could be considered thermal reactions of electronically excited molecules, instead of photoexcitation directly at the VdW and H-bond precursors, photo-to-thermal converters (self-heating molecules or photothermal catalysts) could be applied to generate tuneable thermal energy in the photochemical reaction chamber. For example, through the plasmonic localized heating process, photothermal nanomaterials with high electron mobility, such as Au, Ag, Cu and Al, could transform the absorbed photon energy into highly concentrated thermal energy within 0.1−1.0 ps [36].

## 4. Conclusions

The possibility of the formation of Gly from fundamental gas molecules in cold interstellar media was studied using quantum chemical methods, TST and NVE-MDSH simulations. While previous theoretical studies focused on the multiple pathways to generate Gly in the *S*_0_ state, this study emphasized photochemical pathways in the *S*_1_ state, thermal selectivity and thermodynamic spontaneity after non-radiative *S*_1_→*S*_0_ relaxations, and photo-to-thermal energy conversion in the NVE ensemble.

The optimized bimolecular and termolecular reaction pathways revealed barrierless energies for non-radiative *S*_1_→*S*_0_ relaxations, and the photochemical pathways to generate the HOCOH intermediate from the H_2_O…CO VdW and H_2_O−OC H-bond precursors (Ch (1)_Step (1)) involve considerably lower energy barriers than on the *S*_0_ state pathways and the pathways using the H_2_…CO_2_ VdW precursor (Ch (2)_Step (1)). The optimized reaction pathways also suggested that HCOOH could be formed as an intermediate in Ch (1)_Step (1), and HOCOH could be generated from the *S*_0_→*S*_1_ vertical excitation of HCOOH. For the termolecular reactions, the photochemical pathway that involves the H_2_…CH_2_NH…CO_2_ precursor (Ch (5)) possesses a significantly higher energy barrier than H_2_O…CH_2_NH…CO (Ch (4)) due to the presence of stable molecules (H_2_ and CO_2_).

The TST results suggested that the *T*_s_ for the photochemical pathway using the H_2_O…CO VdW precursor (Ch (1)_Step (1)) is the highest, whereas *T*_s_ for formation of Gly from the HOCOH−CH_2_NH intermediate (Ch (1)_Step (2)) is significantly lower; *T*_s_ is the temperature below which the transition structure is spontaneously formed after the exothermic *S*_1_→*S*_0_ relaxation. The total Gibbs free energy changes (∆*G*˚^,Tot^) confirmed that Ch (1)_Step (2) is thermodynamically more favourable than Ch (1)_Step (1).

To study the photo and thermal selectivity after the exothermic *S*_1_→*S*_0_ relaxation, the NVE-MDSH results on the photochemical pathways in Ch (1), (3) and (4) were subdivided into 15 dynamic reaction pathways. Because all the exothermic *S*_1_→*S*_0_ relaxation temperatures are higher than *T*_s_, almost all the *S*_0_→*S*_1_ vertically excited Wigner-sampled VdW and H-bond precursors in Ch (1)_Step (1) take single-step reaction pathways and return to the precursors, whereas HOCOH and HCOOH could be formed as transient intermediates on consecutive reaction pathways. NVE-MDSH simulations anticipated that in cold interstellar media, without heat exchange between the system and surrounding, formation of Gly is feasible on the photochemical pathways through Ch (1)_Step (1) and Ch (1)_Step (2). Analysis of the representative dynamic reaction pathways revealed the thermal selectivity of molecular processes and products, and the formation of Gly is not high because of the non-synergistic effect of the photochemical and thermochemical pathways.

Because the main drawbacks of photochemical reactions are multiple pathways with unwanted side products, to generate desirable products with high yields, photochemical and thermochemical controlled conditions were suggested as an example. For Gly formation, to alleviate the non-synergistic problem, CO_2_ could be included in the reaction chamber to carry away excess heat and reduce the temperature to below *T*_s_. Alternatively, instead of photoexcitation directly at precursors, a photothermal catalyst could be applied to generate tuneable thermal energy within the reaction chamber; photothermal nanomaterials could transform the absorbed photon energy into highly concentrated thermal energy.

The theoretical results reported in this study confirmed that although quantum chemical and TST methods could give valuable information on the kinetics and thermodynamics of the photochemical and thermochemical pathways, inclusion of the effect of thermal energy through NVE-MDSH simulations is essential if a complete mechanistic study of photochemical processes is to be established.

## Data Availability

The data are provided in the electronic supplementary material [37].
